# Enhancing the *in vitro* and *in vivo* activity of itraconazole against breast cancer using miltefosine-modified lipid nanocapsules

**DOI:** 10.1080/10717544.2021.1917728

**Published:** 2021-05-07

**Authors:** Nabila A. El-Sheridy, Riham M. El-Moslemany, Alyaa A. Ramadan, Maged W. Helmy, Labiba K. El-Khordagui

**Affiliations:** a Department of Pharmaceutics, Faculty of Pharmacy, Alexandria University, Alexandria, Egypt; b European Egyptian Pharmaceutical Industries, Alexandria, Egypt; c Department of Pharmacology, Faculty of Pharmacy, Damanhour University, Damanhour, Egypt

**Keywords:** Itraconazole, miltefosine, lipid nanocapsules, breast cancer, MCF-7 cells, Ehrlich tumor, biomarkers

## Abstract

Itraconazole (ITC), a well-tolerated antifungal drug, exerts multiple anticancer effects which justified its preclinical and clinical investigation as potential anti-cancer agent with reduced side effects. Enhancement of ITC anti-cancer efficacy would bring valuable benefits to patients. We propose herein lipid nanocapsules (LNCs) modified with a subtherapeutic dose of miltefosine (MFS) as a membrane bioactive amphiphilic additive (M-ITC-LNC) for the development of an ITC nanoformulation with enhanced anticancer activity compared with ITC solution (ITC-sol) and unmodified ITC-LNC. Both LNC formulations showed a relatively small size (43–46 nm) and high entrapment efficiency (>97%), though ITC release was more sustained by M-ITC-LNC. Cytotoxicity studies revealed significantly greater anticancer activity and selectivity of M-ITC-LNC for MCF-7 breast cancer cells compared with ITC-sol and ITC-LNC. This trend was substantiated by *in vivo* findings following a 14 day-treatment of murine mammary pad Ehrlich tumors. M-ITC-LNC showed the greatest enhancement of the ITC-induced tumor growth inhibition, proliferation, and necrosis. At the molecular level, the tumor content of Gli 1, caspase-3, and vascular endothelial growth factor verified superiority of M-ITC-LNC in enhancing the ITC antiangiogenic, apoptotic, and Hedgehog pathway inhibitory effects. Finally, histopathological and biochemical analysis indicated greater reduction of ITC systemic toxicity by M-ITC-LNC. Superior performance of M-ITC-LNC was attributed to the effect of MFS on the structural and release properties of LNC coupled with its distinct bioactivities. In conclusion, MFS-modified LNC provides a simple nanoplatform integrating the potentials of LNC and MFS for enhancing the chemotherapeutic efficacy of ITC and possibly other oncology drugs.

## Introduction

Itraconazole (ITC) is a well-tolerated fungicidal triazole compound (Houšť et al., 2020). In recent years, preclinical and clinical evidence verified ITC activity against different cancers, rationalizing its potential repurposing as chemotherapeutic agent (Tsubamoto et al., 2017; Wang et al., 2017b). ITC acts mainly by inhibiting the Hedgehog (Hh) pathway (Wei et al., 2020), tumor growth and angiogenesis and by inducting apoptosis and autophagy (Li et al., 2019; Wei et al., 2020). ITC also inhibits the P-glycoprotein efflux pump, reversing chemoresistance (Correia et al., 2018; Elmeliegy et al., 2020). Prospective clinical trials of different cancers including prostate cancer, basal cell carcinoma, ovarian cancer, and triple-negative breast cancer documented clinical benefits of ITC (Tsubamoto et al., 2017).

Different approaches were demonstrated to enhance the anticancer activity of ITC, particularly combinatorial therapy with oncology drugs (Correia et al., 2018; Sawasaki et al., 2020) and nanocarrier-mediated delivery (Correia et al., 2018; Alhakamy & Md, 2019). The latter approach offers a wide range of benefits, notably masking poor drug solubility, controlling drug release, and enhancing intracellular delivery (Aghebati-Maleki et al., 2020). Combining both approaches could further enhance ITC activity. For instance, nanocarriers combining ITC and different oncology drugs demonstrated greater activity relative to the singly loaded formulations (Okeke et al., 2017; Lin et al., 2018; Zhang et al., 2018). Furthermore, coating of ITC nanocarriers with additives such as Pluronic 123 (Lin et al., 2018) and didodecyldimethylammonium bromide (Carbone et al., 2018) was also reported to enhance ITC chemotherapeutic activity. We propose herein lipid nanocapsules (LNCs) modified with a membrane active additive, miltefosine (MFS), as a new potential ITC repurposing formulation with enhanced anticancer activity. The proposed formulation integrates the biopharmaceutical advantages of LNC in delivering oncology drugs and the structural and membrane active properties of MFS in favor of ITC anticancer activity.

LNC are physically stable bio-inspired lipoprotein-like nanovectors characterized by a small uniform size (less than 100 nm) and an oily core surrounded by a pegylated surfactant tensioactive shell (Huynh et al., 2009). LNC were reported to improve the therapeutic profile of anti-cancer drugs (Resnier et al., 2017; Fourniols et al., 2020), enhance tumor localization and intracellular drug delivery (Huynh et al., 2011; Karim et al., 2018) and inhibit the P-glycoprotein efflux pump (Garcion et al., 2006). MFS, an amphiphilic membrane active alkylphospholipid (APL) synthetically derived from cell membrane components, shows multiple biological actions including anticancer activity (Kaleağasıoğlu et al., 2019, 2020). MFS is currently approved for the oral treatment of leishmaniasis (50 mg twice or thrice daily) and topical treatment (6% solution) of breast cancer skin metastasis. We demonstrated earlier that MFS in a subtherapeutic dose integrates within the oil core/pegylated surfactant shell interface of LNC, enhancing their structural integrity and biopharmaceutical performance (Eissa et al., 2015). This also resulted in enhanced hemocompatibility and bioactivity of MFS (Eissa et al., 2015, 2020). As an APL compound, MFS exerts anticancer activity by acting on cell membranes rather than DNA (van Blitterswijk & Verheij, 2013), interfering with lipid metabolism and survival signaling pathways (Kaleağasıoğlu et al., 2019; Zulueta Diaz et al., 2020). ALP compounds also induce cell cycle arrest and autophagy via inhibition of the Akt/mTOR cascade (Kaleağasıoğlu et al., 2020) and are most promising in combination with anticancer drugs (Yosifov et al., 2014; Uzunova et al., 2019).

The objective of the current study was to utilize MFS-modified LNC (M-ITC-LNC) incorporating MFS (0.06%) as a new bioactive nanocarrier to enhance the anticancer activity of ITC. Such a system would provide multiple advantages including MFS-induced enhancement of the structural integrity of ITC-LNC, sustainment of ITC release and improvement of the anticancer activity of ITC via MFS membrane activity and an MFs/ITC combinational effect. The efficacy of the proposed M-ITC-LNC relative to ITC solution (ITC-sol) and unmodified ITC-LNC was assessed against breast cancer. The cytotoxicity and selectivity of the test formulations were appraised using human adenocarcinoma MCF-7 breast cancer cells and normal human fibroblasts. Their antitumor efficacy and systemic toxicity were determined utilizing a murine Ehrlich ascites breast cancer model and doxorubicin (DOX) for comparison. Assessments included % change in tumor weight and volume alongside tumor proliferation and necrosis determined by histopathological and immunohistopathological examination. The tumor content of the biomarkers, Gli 1, caspase-3, and vascular endothelial growth factor (VEGF), was determined to assess the effect of test LNC formulations on the anticancer effects of ITC. Systemic toxicity was assessed biochemically and by histopathological examination of the liver and kidney of mice post treatment.

## Materials and methods

### Materials

Itraconazole was purchased from Neuland Laboratories Limited (Telangana, India), >99% and MFS (1-hexadecylphosphocholine) was purchased from Chem-Impex International (New York, NY), 98–100%. Labrafac™ lipophile WL 1349 (Labrafac, caprylic-capric acid triglycerides, European Pharmacopeia, IVth, 2002), Labrafil^®^ M1944 CS (oleoyl polyoxyl-6 glycerides), and Transcutol HP^®^ (diethylene glycol monoethyl ether), were gift of Gattefossé S.A. (Saint-Priest, France). Lipoid^®^ S75-3 (soybean lecithin at 69% phosphatidylcholine and 10% phosphatidyl ethanolamine, average MW 800) was purchased from GMBH (Ludwigshafen, Germany). Kolliphor^®^ HS 15 (Solutol^®^ HS-15) was purchased from BASF (Ludwigshafen, Germany). Sodium lauryl sulfate (SLS), ethylene diamine-tetraacetic acid (EDTA), hematoxylin and eosin (H&E) solutions, 3-(4,5-dimethylthiazolyl-2)-2,5-diphenyltetrazolium bromide (MTT), and 1,1′-dioctadecyl-3,3,3′,3′-tetramethylindocarbocyanine perchlorate (DiI) were purchased from Sigma-Aldrich (St. Louis, MO). Fetal bovine serum (FBS) was purchased from GIBCO^®^ Life Technologies (Carlsbad, CA). Methanol, acetonitrile, and dimethyl sulfoxide were purchased from Fisher Scientific (Waltham, MA).

### Formulation of lipid nanocapsules

Blank LNC were prepared by the phase inversion method (Heurtault et al., 2002) with modification. The LNC formulation ingredients in % w/w of the final dispersion weight were mixed in a closed container under magnetic stirring. These included Labrafil (10%), Kolliphor^®^ HS 15 (Kolliphor) (10%), Transcutol (7%), sodium chloride (1%), Lipoid (1.5%), and demineralized water (20.5%). The mixture was subjected to three cycles of progressive heating and cooling between 60 and 90 °C at a rate of 4 °C/min with the formation of a w/o emulsion and a o/w emulsion, above and below the phase inversion temperature, respectively. An irreversible shock was induced by a twofold dilution with deionized cold water (0–2 °C) added at a temperature 1–3 °C from the beginning of the phase inversion zone. The LNC dispersion was stirred slowly using a magnetic stirrer for 5 min and kept at 4 °C. For the preparation of ITC-LNC, the same procedure was adopted following addition of ITC to the oil phase in a 0.3% w/w concentration. For M-ITC-LNC, ITC (0.3% w/w) and MFS (0.06% w/w), at a ratio of 5:1 by weight, were included in the oil phase. Blank fluorescent LNC labeled with the DiI dye (DiI-LNC) were prepared as reported (Morille et al., 2010). Briefly, a stock solution of Dil (0.6% w/w) was prepared in acetone. The solvent was then evaporated at 80 °C and the dye residue dissolved in Labrafil. The initial ratio of the stock Dil solution to Labrafil was 1:10 by weight. LNCs were prepared as described above.

### *In vitro* characterization of lipid nanocapsules

#### Physical properties, transmission electron microscopy (TEM), and entrapment efficiency (EE%)

The mean hydrodynamic diameter and polydispersity index (PDI) of LNC were determined by dynamic light scattering (DLS) using Malvern Zetasizer^®^ at a fixed angle (173°) at 25 °C and a 4 mW He–Ne laser at 633 nm (Zetasizer^®^ Nano ZS series DTS 1060, Malvern Instruments S.A., Worcestershire, UK). Zeta potential was determined at 25 °C in water (dielectric constant 79, refractive index 1.33, viscosity 0.89 cP) using a cell voltage of 150 V and 5 mA current. The morphology of ITC-LNC and M-ITC-LNC was examined by TEM using JEOL, JEM-100 CX Electron Microscope (Tokyo, Japan). Before analysis, LNC dispersions were sprayed onto copper grids and stained with 2% w/v uranyl acetate solution. Shots were taken at ×50k at 80 kV. Entrapment efficiency was calculated based on the difference between the amounts of entrapped and un-entrapped ITC. LNC were separated using an ultrafiltration/centrifugation technique and ITC was assayed in the ultrafiltrate by an isocratic reverse phase HPLC method with UV detection at 225 nm (El-Sheridy et al., 2019). Briefly, separation was performed on Inertsil C-18 column (ODS, 250 × 4.6 mm, 5 μ). The mobile phase was a mixture of tetrabutylammonium hydrogen sulfate buffer (2.72% w/v) and acetonitrile (40:60) flowing at a rate of 1.5 mL/min. The injection volume was 20 μL and retention time 3.5–4 min. All measurements were done in triplicate under ambient conditions. ITC EE% and payload were calculated as follows:

$$\text{EE}\%=\frac{\text{total}\;\text{drug}\;\text{content}\;(\text{mg})-\text{unentrapped}\;\text{drug}\;(\text{mg})}{\text{total}\;\text{drug}\;\text{content}\;(\text{mg})}\times \;100$$

$$\text{ITC}\;\text{payload}\;=\;\frac{\text{entrapped}\;\text{drug}\;(\text{mg})}{\text{LNC}\;\text{dry}\;\text{weight}\text{ (g)}}$$

#### Solid state properties

The thermal behavior of ITC and MFS alone and in freeze-dried LNC formulations was examined by differential scanning calorimetry (DSC) using a DSC-6 differential Scanning Calorimeter (PerkinElmer Instruments, Waltham, MA). DCS traces were recorded between 40 and 400 °C at a constant 20 °C/min rate under an atmosphere of nitrogen purged at a flow rate of 20 mL/min. An empty pan was used as reference. Fourier transform infrared (FT-IR) spectra of the same samples were recorded from 4000 to 500 cm^−1^ using FT-IR Spectrometer (PerkinElmer Instruments, Waltham, MA) after compression of the samples with IR grade KBr into discs.

#### Itraconazole release

ITC release from M-ITC-LNC in comparison with ITC-LNC was investigated at 37 ± 0.5 °C in a thermostatically controlled shaking water bath at 100 rpm. A developed release medium allowing sink conditions was based on a solubility study of ITC in phosphate buffer saline (PBS, pH 7.4) containing SLS in increasing concentrations. A known volume of the LNC dispersion was added to 5 mL of the selected release medium (5% SLS in PBS pH 7.4) in closed flasks. At different time intervals, LNC were separated by ultracentrifugation at 12,000 rpm for 10 min at 4 °C using Vivaspin^®^6 centrifugal filters. The filtrate containing the released ITC was injected into the HPLC column. Results expressed as cumulative ITC release (%) are the average of three determinations. The release kinetics of ITC-LNC were determined by fitting release data to different mathematical models and choosing the best fit by regression analysis using an Excel add-in.

#### Hemocompatibility

The hemocompatibility of M-ITC-LNC in comparison with ITC-sol in DMSO and ITC-LNC was assessed by evaluating their *in vitro* erythrocyte hemolytic activity using fresh human blood (Eissa et al., 2015). In brief, 2 mL blood were collected from a young healthy volunteer under medical supervision after obtaining approval of the research ethics committee of the Faculty of Pharmacy, Alexandria University (approval no. 200501) based on informed consent. The blood sample was mixed with EDTA and centrifuged at 2500 rpm for 10 min. RBCs were washed three times with PBS pH 7.4 and diluted to a 1% v/v hematocrit suspension using PBS. Samples of ITC-sol or LNC dispersions equivalent to 3 mg/mL and 0.3 mg/L ITC were mixed with 2 mL of the RBCs suspension and the mixtures incubated for 45 min at ambient temperature (≈25 °C). Optical density of the supernatants was measured at 540 nm following centrifugation at 2500 rpm for 10 min. Positive and negative controls were obtained by incubating the hematocrit suspension with 0.1% w/v Triton X-100 and PBS, respectively under the same conditions. The % hemolysis was calculated as follows:

$$\text{\% Hemolysis }=\;\frac{\;(\mathrm{At}-\mathrm{An})}{\;(\mathrm{Ap}-\mathrm{An})}\times 100\%$$

where At is the absorbance value of the test sample, An and Ap are the absorbance values of the negative and positive controls, respectively.

#### Cytotoxicity and selectivity for cancer cells

The cytotoxicity of M-ITC-LNC in comparison with ITC-sol and ITC-LNC at different ITC concentrations on human breast adenocarcinoma MCF-7 cell line (American Type Culture Collection (ATCC), Manassas, VA) was evaluated in triplicate using the MTT assay. DMSO and blank LNC were used as controls. Cells were maintained in Dulbecco’s modified Eagle medium (DMEM) containing 10% FBS in a CO_2_ incubator (5% CO_2_ at 37 °C) and were then seeded (5 × 10^3^/well) in a 96-well plate containing 100 μL of DMEM and allowed to adhere to the plate for 24 h. The medium was replaced with a fresh medium containing ITC-sol, ITC-LNC or M-ITC-LNC and incubated for another 24 h. The cells were washed twice with PBS pH 7.4 after removal of the treatments and were incubated with 100 μL MTT solution (0.5 mg/mL in DMEM) for further 4 h at 37 °C in the dark. After removal of the supernatant by centrifugation at 2000 rpm for 10 min, 100 μL of DMSO was added to the wells to dissolve the MTT-formazan crystals by agitation for 15 min. Absorbance was measured at 570 nm using a microplate reader (Model 550, Bio-Rad, Hercules, CA). Values for IC50 were determined using Origin 8.0 software (Origin Lab, Northampton, MA). Dose–response curves were plotted after correction by subtracting the background absorbance from the controls. The relative cell viability (%) was calculated relative to the untreated control cells as follows:

% Cell viability=A/Ac×100

where *A* is the absorbance of the treated wells and Ac is the absorbance of control wells. Values for IC50 were extrapolated graphically from the plotted data using a polynomial regression equation.

The effect of M-ITC-LNC in comparison with ITC-sol and ITC-LNC on normal cells was assessed using human fibroblast cells. Systems containing ITC-LNC dispersions or ITC-sol in increasing concentrations were incubated with the cells (5 × 10^3^ cells/well) that were cultured in DMEM containing 5% FBS. Following 24 h incubation, the % cell viability was calculated using the MTT assay and the IC50 values for normal cells estimated. Selectivity for MCF-7 cancer cells was assessed by calculating the selectivity index (SI) using the following equation (Rashidi et al., 2017):

$$\mathrm{SI}=\;\frac{\;\mathrm{IC}50\text{ in normal cell line}}{\mathrm{IC}50\text{ in cancer cell line}}$$

#### *In vivo* studies

*In vivo* studies were performed on mice according to the ethical guidelines of Alexandria University which comply with the National Institutes of Health guide for the care and use of Laboratory animals (NIH Publications No. 8023, revised 1978). The study protocol was approved by the Institutional Animal Care and Ethics Committee of the Faculty of Pharmacy, Alexandria University, Egypt (approval code 06-277-2020). A total of 30 female BALB/c mice 7–8 weeks old and weighing 20–25 g were randomly housed at 22 ± 5 °C in a 12 h light/dark cycle at 6 mice/cage. Mice were fed rodent chow and water at libitum. *In vivo* studies were conducted for visualizing the biodistribution of fluorescent blank DiI-LNC and assessment of the antitumor efficacy and systemic toxicity of M-ITC-LNC relative to ITC-sol and ITC-LNC using DOX for comparison. Mice were weighed at baseline and 7- and 14-days post treatment initiation.

#### Biodistribution of blank LNC

Biodistribution of LNC was assessed by fluorescence imaging of DiI-LNC (Morille et al., 2010) using untreated mice as control. Test mice were injected intraperitoneally (i.p.) with 100 μL DiI solution or DiI-LNC dispersion having a similar DiI concentration. Mice were sacrificed 6 h post treatment with a large dose of thiopental (50 mg/kg. i.p.) and their main organs; liver, spleen, kidneys, and heart collected for imaging. Fluorescent signals were visualized at emission wavelength 549 nm and excitation wavelength 565 nm (PhotonIMAGER^TM^ Optima, Biospace Lab, Nesles-la-Vallée, France).

#### Anti-tumor efficacy

Mammary tumors were induced in female balb/c mice using Ehrlich ascites tumor (EAT) cells obtained from the National Cancer Institute, Cairo, Egypt. In brief, approximately 10^7^ of EAT cells suspended in PBS, were inoculated subcutaneously into the left side of the mammary fat pad of mice (Elzoghby et al., 2017). Treatment started when the solid tumors became visible (50–100 mm^3^) 14 days post cell inoculation and continued for 14 days. Tumor-bearing mice were randomly divided into five groups, six mice each, as follows: group 1: control (untreated), group 2: DOX injection (Dox, 5 mg/kg once weekly), group 3: ITC-sol in DMSO (10 mg/kg once daily), group 4: ITC-LNC (10 mg/kg once daily), and group 5: M-ITC LNC providing the dose of 10 mg/kg ITC and 2 mg/kg MFS, once daily. At the end of the 14 day-study, animals were sacrificed with a large dose of thiopental (50 mg/kg. i.p.) and the excised tumors were washed with ice-cold phosphate buffer and their weights determined. Tumors were then divided into portions for different assessments. Antitumor efficacy was assessed by determining the % change in tumor volume and tumor weight, histopathological and immunohistopathological examination of the tumor for necrotic and antiproliferative effects, respectively, alongside quantitative determination of three tumor biomarkers.

#### Tumor growth

The % change in tumor volume in all mice was determined at days 7 and 14 from the start of treatment compared to baseline tumor volume. Tumor volume was calculated by measuring the tumor length (major axis) and width (minor axis) with a Vernier caliper as follows:

Tumor volume= 43 π (minor axis)2 ×major axis

#### Tumor necrosis

Tumor necrosis was examined histopathologically. Sections of the fixed excised mammary tumors, 5 μm thick, were stained with H&E, dehydrated in alcohol, mounted in Canada balsam, and examined blindly by optical microscopy. Necrosis was assessed semi-quantitatively as the ratio of necrosis area to the total area in 10 random sections of each tumor using a scoring scale based on % necrosis in sections in poorly differentiated tumor (Elzoghby et al., 2017).

#### Tumor proliferation

The anti-proliferative activity of test formulations was determined by tumor immunohistochemical staining of the Ki-67 proliferating protein in formalin-fixed, paraffin-embedded specimens (Yi et al., 2018). Briefly, 4 μm-thick tumor tissue sections were dried, deparaffinized, and rehydrated following standard procedures. Sections were subjected to heat-induced antigen retrieval. Immunohistochemical staining was performed using Ki-67 antibody (Monoclonal Mouse Anti-Human, Dako Agilent autostainer (Twinsburg, OH)). Optical densities of the Ki 67-positive areas in the tumor sections were measured and the % Ki-67 proliferative protein expression calculated. This was achieved by digital image analysis using Image J software (version 1.45s, Bethesda, MD) together with computer-assisted microscopy (El Sayed et al., 2018).

#### Tumor biomarkers

Three tumor biomarkers, glioma-associated oncogene 1 (Gli 1), caspase-3, and VEGF were determined quantitatively in samples of excised tumors using ELISA Kits (MyBioSource Inc., San Diego, CA) according to the respective manufacturer’s protocols.

#### Toxicity study

Systemic toxicity of the test ITC-based formulations was evaluated by monitoring animal weight at day 7 and day 14, histopathological examination of the mice livers and kidneys and biochemical analysis of the liver and kidney functions. At the end of the *in vivo* study and before animal sacrifice, blood was collected by aortic vein puncture in microcentrifuge tubes and centrifuged at 2500 rpm for 5 min at 4 °C. The supernatant serum was assayed for alanine transaminase (ALT), aspartate aminotransferase (AST), blood urea nitrogen (BUN), and serum creatinine levels using commercial kits (Accurex^®^). In parallel, biopsies of the livers and kidneys of sacrificed mice were collected, fixed in 10% neutral buffered formalin, and embedded in paraffin for histopathological examination. Sections were stained with H&E and digital shots were taken.

### Statistical analysis

Data were analyzed using Minitab ver. 17 software. Data expressed as means ± SD are representative of at least three measurements. The differences between multiple groups were assessed by one-way analysis of variance (ANOVA) and Dunnett’s test. A value of *p* < .05 was indicative of significance.

## Results and discussion

### Formulation of lipid nanocapsules

The development of M-ITC-LNC <100 nm in diameter and having high EE% and relatively high ITC payload was guided by earlier studies involving different applications (Eissa et al., 2015; El-Sheridy et al., 2019). LNC are conventionally formulated with Labrafac as the main oil core component and Kolliphor^®^ HS 15 as the pegylated surfactant forming the tensioactive shell (Heurtault et al., 2002; Huynh et al., 2009). In a series of single point preliminary experiments, a combination of Labrafac and Kolliphor allowed for a maximum ITC payload of 1.83 mg/g. Transcutol which increases the solubility of ITC (Choi et al., 2012) was then used as a co-surfactant in combination with Kolliphor. This necessitated replacement of Labrafac with the more hydrophilic Labrafil as the oil core in order to enhance the incorporation of Transcutol (Roger et al., 2011). Finally, a formulation based on 10% w/w Labrafil, 10% Kolliphor, and 7% w/w Transcutol allowed the incorporation of 0.3% ITC at 10.2 mg/g payload. MFS could be added to this formulation in a 0.06% w/w concentration. The developed ITC-LNC and M-ITC-LNC formulations were used in subsequent studies.

### Characteristics of lipid nanocapsules

#### Physical properties, TEM, and entrapment efficiency

**Figure 1.**
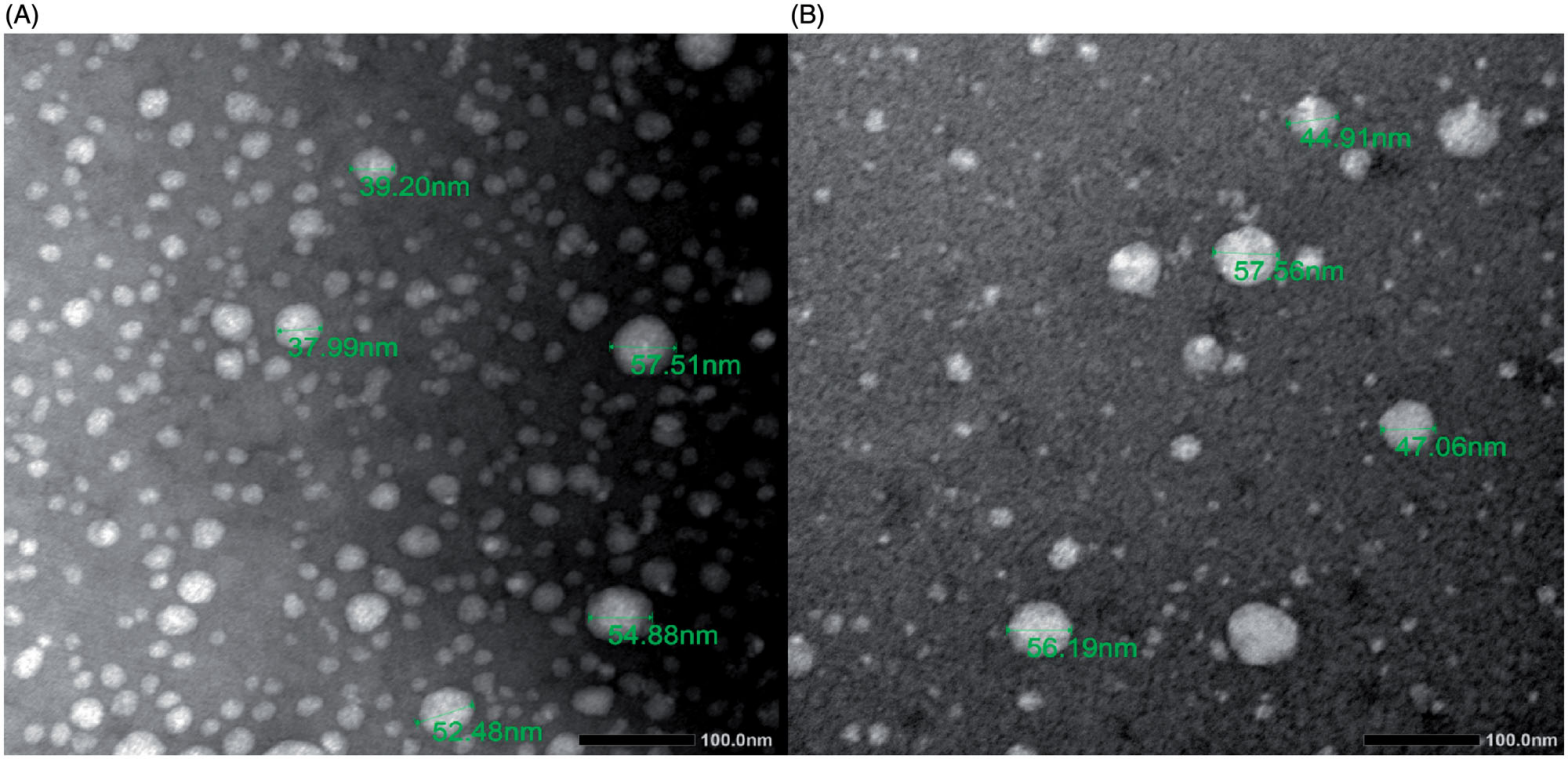
TEM of (A) ITC-LNC and (B) M-ITC-LNC.

LNC generally showed a relatively small size (43.1–46.0 nm) and PDI (0.18–0.24) which were not affected by ITC loading (Table 1). For lipid-based nanocarriers, a PDI of 0.3 and below indicates a homogenous population (Danaei et al., 2018). The moderately negative ZP of LNC (–13.4 to –22.3 mV) can be attributed to the hydrolysis of a small fraction of surfactants, leading to negatively charged groups (Mouzouvi et al., 2017). Noteworthy, surface charge is not the sole factor affecting colloidal stability of LNC as the tensioactive surface layer plays a significant role in this respect (Roger et al., 2011). TEM (Figure 1) indicated that ITC-LNC and M-ITC-LNC showed a nearly similar size and were almost spherical, homogenously distributed and not aggregated. ITC-LNC formulations showed EE% exceeding 97%. MFS was reported earlier to integrate efficiently within LNC (>97%) most probably at the core/surfactant shell interface with the hydrophobic alkyl chain extending into the lipid core and the zwitter ionic group directed outwards, increasing the LNC structural integrity (Eissa et al., 2015). A similar orientation of MFS in polymeric micelles has been demonstrated (Puig-Rigall et al., 2020).

**Table 1.** LNC formulations and their physical properties.

LNC formulations	Size, nm	PDI	ZP, mV
Blank LNC	43.1 ± 1.4	0.23 ± 0.02	–13.4 ± 0.5
ITC-LNC	46.0 ± 0.5	0.24 ± 0.06	–18.3 ± 3.4
M-ITC-LNC	43.2 ± 0.6	0.18 ± 0.02	–22.3 ± 1.9

Data are the mean ± SD of at least three experiments.

#### Solid state properties

**Figure 2.**
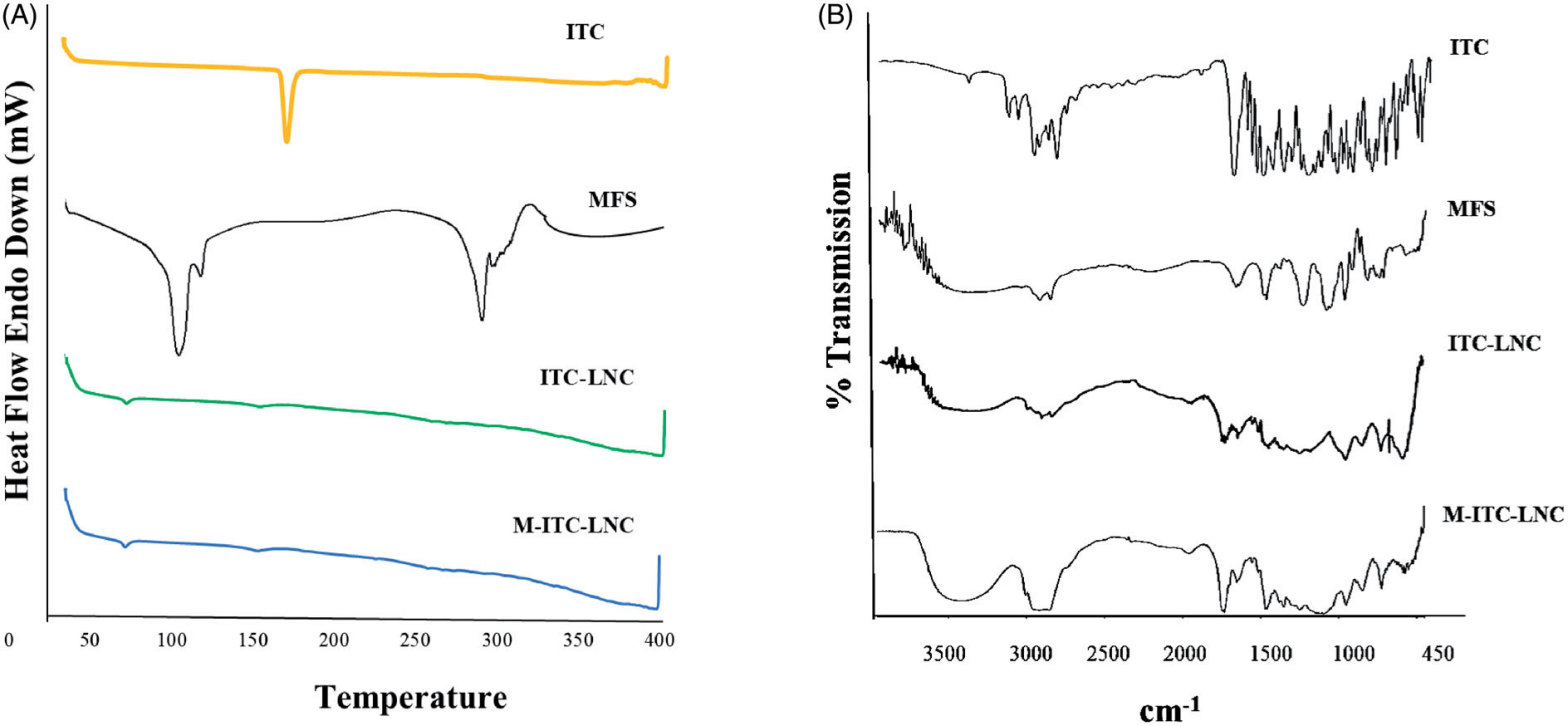
Solid state properties of ITC, MFS, ITC-LNC, and M-ITC-LNC: (A) DSC and (B) FT-IR scans.

DSC thermograms and FT-IR spectra of ITC, MFS and lyophilized ITC-LNC and M-ITC-LNC are shown in Figure 2(A,B), respectively. DSC scan for ITC showed a sharp melting endotherm at 169.5 °C while MFS scan showed a peak at 95.4 °C attributed to loss of water of hydration and a sharp melting endotherm at 261.8 °C. Disappearance of the ITC and MFS melting peaks in the thermograms of lyophilized ITC-LNC and M-ITC-LNC indicated molecular dispersion of ITC into the LNC. The FT-IR spectrum of ITC showed characteristic absorption bands between 2800 and 3400 cm^−1^ attributed to the aromatic CH and NH_2_ groups, peaks at 1613 cm^−1^ and 1425 cm^−1^ due to the C=N and C–N bonds, respectively, and a sharp peak at 1699 cm^−1^ ascribed to the stretching of the C=O bond (Feng et al., 2018). On the other hand, the MFS spectrum showed characteristic peaks at 2950–2850 cm^−1^ and 1473 cm^−1^ corresponding to CH_2_ stretching and CH_2_ bending, respectively. The spectrum also showed a peak at 1246 cm^−1^ due to P=O asymmetric stretching and a broad peak, mostly corresponding to the P–O–C bond at ∼1050 cm^−1^ (Dorlo et al., 2012). Spectra of ITC-LNC and M-ITC-LNC indicated preservation of most of the ITC and MFS characteristic peaks.

#### Itraconazole release

**Figure 3.**
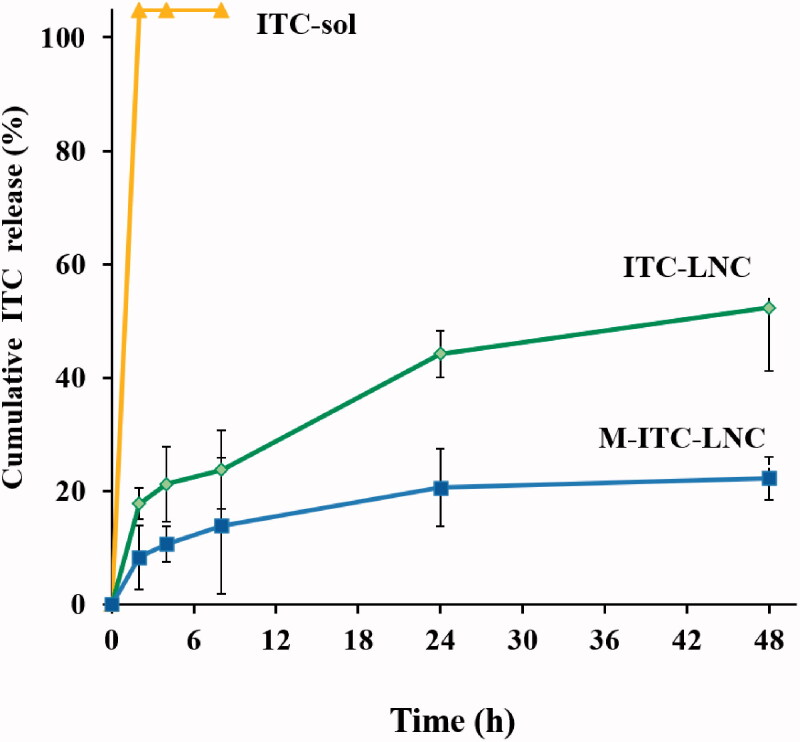
ITC release from lipid nanocapsule formulations in comparison with free ITC in PBS pH 7.4/5% sodium lauryl sulfate medium at 37 °C and 100 rpm. Results are means ± SD (*n* = 3).

A medium allowing sink conditions for ITC release at physiological pH at 37 °C was developed using PBS pH 7.4 containing 5% SLS. ITC solubility in the medium at 37 °C was 0.317 ± 0.01 mg/mL. Free ITC dispersed in this medium at 37 °C and 100 rpm was rapidly dissolved, verifying sink conditions (Figure 3). Lipid nanoencapsulation led to sustained release of ITC according to a parabolic release profile with a limited burst effect. ITC release from ITC-LNC attained 52.2% in 48 h and was significantly (*p* < .05) sustained (22.5%) by M-ITC-LNC. As lipophilic drugs are released from LNC via diffusion into the aqueous medium across the tensioactive interfacial barrier (Abdel-Mottaleb et al., 2010), significant reduction of ITC release by M-LNC confirmed increased resistance of the LNC tensioactive core/pegylated surfactant shell to ITC release as a result of incorporation of the amphiphilic MFS molecules into the shell. MFS was also reported to sterically stabilize other lipid based systems such as liposomes (Arndt et al., 1997). Greater structural integrity of M-ITC-LNC and sustained release of ITC are beneficial to the *in vivo* performance of these nanocarriers. Analysis of ITC release data over 48 h according to different kinetic models indicated Weibull and Korsmeyer–Peppas kinetics. The Weibull shape parameter (*β*) was less than 1, denoting a case 3 parabolic release profile (Zhang et al., 2010) which described the ITC release profiles. Fitting data to the Korsmeyer–Peppas model showed *n* values less than 0.5, indicating Fickian diffusion-controlled ITC release.

#### Hemocompatibility

**Figure 4.**
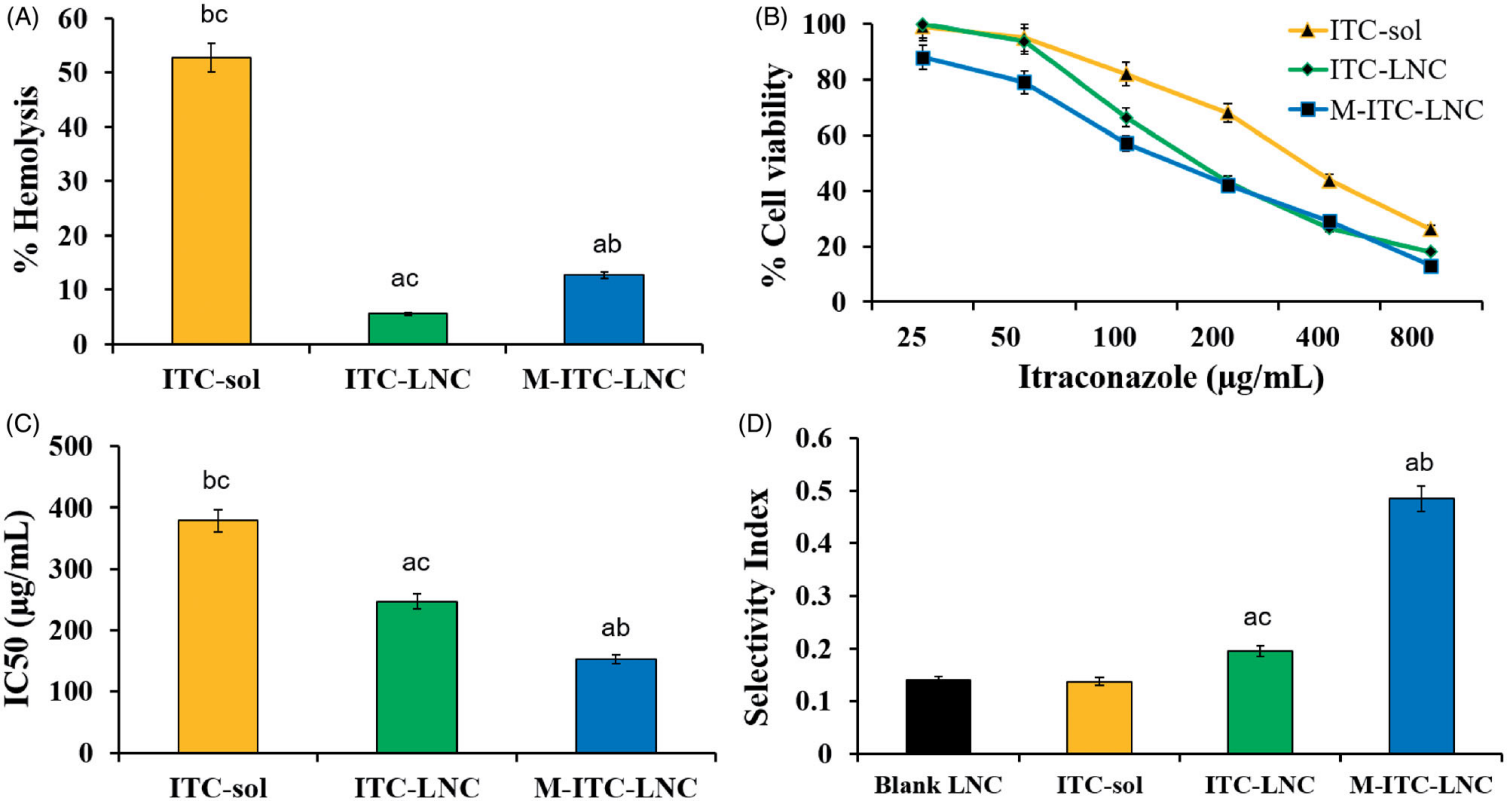
Bioactivity of ITC test formulations (A) hemolytic activity; (B) MCF-7 cell viability curves, (C) derived IC50 values, and (D) selectivity index (SI) determined using normal human fibroblasts. Error bars represent SD (*n* = 3). ^a^*p*<.05 vs. ITC-sol, ^b^*p*<.05 vs. ITC-LNC, and ^c^*p*<.05 vs. M-ITC-LNC.

*In vitro* hemolysis of human erythrocytes by M-ITC-LNC in comparison with ITC-sol in DMSO and ITC-LNC at a drug concentration of 3 mg/mL was used as surrogate indicator of hemocompatibility. As shown in Figure 4(A), incubation of RBCs with ITC-sol for 45 min induced 52.8% hemolysis, mostly due to ITC since DMSO-induced hemolysis was 5.29%, in accordance with reported data (de Abreu Costa et al., 2017). Lipid nanoencapsulation significantly (*p* < .05) reduced the hemolytic activity of ITC to ≈6% due to the dense protective Lipoid/Kolliphor^®^ stealth shell of LNC. Stealth nanocarriers hinder interaction with the erythrocyte membrane, enhancing hemocompatibility of the entrapped drug (El-Lakany et al., 2018). The protective effect of LNC against ITC-induced hemolysis was slightly but significantly reduced (*p* < .05) by MFS modification. Free MFS is known to induce hemolysis by destabilization of the erythrocyte membrane (Alonso et al., 2019). However, the hemolytic activity of MFS decreases significantly by LNC (Eissa et al., 2015). It is worth noting that dilution of ITC-LNC and M-ITC-LNC dispersions to attain an ITC concentration of 0.3 mg/mL to be adopted in subsequent *in vivo* studies significantly (*p* < .05) reduced hemolysis to less than 5%, implying hemocompatibility of both formulations.

#### Cytotoxicity and selectivity for cancer cells

The cytotoxicity of M-ITC-LNC in comparison with ITC-sol and ITC-LNC over an ITC concentration range of 25–800 µg/mL was assessed using MCF-7 cell line following 24 h contact with the cells at 37 °C. Results for cell viability and IC50 are shown in Figure 4(B,C), respectively. DMSO and blank LNC, used as controls, showed relatively low cytotoxicity with IC50 of 1160 µg/mL and 1880 µg/mL, respectively. Blank LNC exert a different degree of cytotoxicity against different cell lines but MCF-7 cells show low sensitivity to LNC (Szwed et al., 2020). On the other hand, ITC-sol was cytotoxic to MCF-7 cells in a concentration-dependent manner with an IC50 of 378.7 ± 19.8 µg/mL (Figure 4(C)). ITC was reported to dramatically reduce MCF-7 cell viability and significantly induce cell death via apoptosis mainly due to alteration of mitochondria membrane potential, reduction of Bcl-2 expression and increase of caspase-3 activity (Wang et al., 2017b). LNC significantly (*p* < .05) reduced the IC50 of ITC-sol (1.5-fold) (Figure 4(C)). Such a potentiating effect can be attributed to the small size, biomimicry, and cellular interactions of LNC, all enhancing intracellular drug delivery (Karim et al., 2018; Lollo et al., 2019). Notably, M-ITC-LNC reduced the IC50 of ITC-sol by 2.5-fold, indicating a significant increase in anticancer activity of ITC-LNC via MFS modification. Increased LNC integrity and sustained ITC release in addition to the bioactivity of MFS may account for anticancer activity enhancement. MFS was reported to enhance the cytotoxicity of anticancer drugs whether in a combination (Yosifov et al., 2014; Uzunova et al., 2019) or conjugated form (Zhou et al., 2019). Selectivity of the test formulations for cancer cells relative to normal cells was expressed as the SI (Figure 4(D)). Lipid nanoencapsulation significantly (*p* < .05) increased the selectivity of ITC for MCF-7 cells (1.4-fold). M-ITC-LNC achieved a further significant (*p* < .05) selectivity enhancement (3.5-fold). Results implied substantial reduction of indiscriminative cytotoxicity and greater selectivity of ITC treatment by MFS-modified LNC.

### *In vivo* studies

#### Biodistribution blank LNC

**Figure 5.**
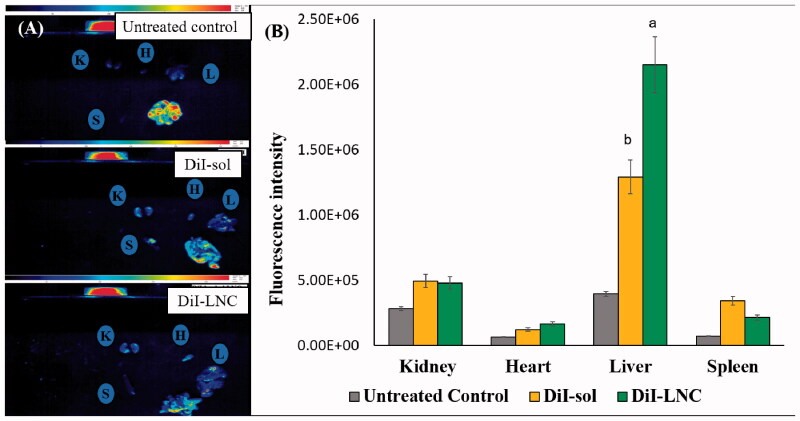
Biodistribution of DiI-LNC in comparison with DiI solution and untreated control. (A) Fluorescent luminescent images of organs isolated 6 h post intraperitoneal administration (K: kidney, H: heart, L: liver, S: spleen) and (B) fluorescence intensity in different organs. Error bars represent SD (*n* = 3). ^a^*p*<.05 vs. Dil-sol, ^b^*p*<.05 vs. Dil-LNC.

Biodistribution of blank DiI-LNC in comparison with Dil-sol was examined to verify the structural stability and accumulation of LNC in vital organs following i.p. injection. Dil is a lipophilic indocarbocyanine dye proven to be retained in LNC (Ballot et al., 2006; Hirsjarvi et al., 2013). Distribution of Dil-LNC in comparison with Dil-sol to the liver, spleen, kidneys, and heart of mice, 6 h post injection was expressed as fluorescence intensity (Figure 5). DiI-sol accumulated predominantly in the liver and to a lesser extent the kidneys, spleen, and heart while Dil-LNC were more concentrated in the liver (1.67-fold). This can be attributed to the inherent uptake of LNC by the liver and to a lesser extent the kidneys and heart (Ballot et al., 2006; Hirsjarvi et al., 2013). Results supported structural integrity and organ distribution of LNC.

#### Anti-tumor efficacy

The Ehrlich ascites mammary tumor model utilized in the present study is a transplantable rapidly growing undifferentiated carcinoma model for breast cancer showing sensitivity to chemotherapy (Ozaslan et al., 2011). Efficacy assessment of the test formulations started following administration of the equivalent of 10 mg/kg ITC as ITC-LNC or 10 mg/kg ITC + 2 mg/kg MFS as M-ITC-LNC daily by i.p. injection for 14 days. ITC dose was in the range of anticancer ITC dosing in mice, 8–130 mg/kg (Wang et al., 2015). Dox, a potent chemotherapeutic agent against breast cancer, was used weekly in a 5 mg/kg dose for comparison.

#### Tumor growth inhibition

**Figure 6.**
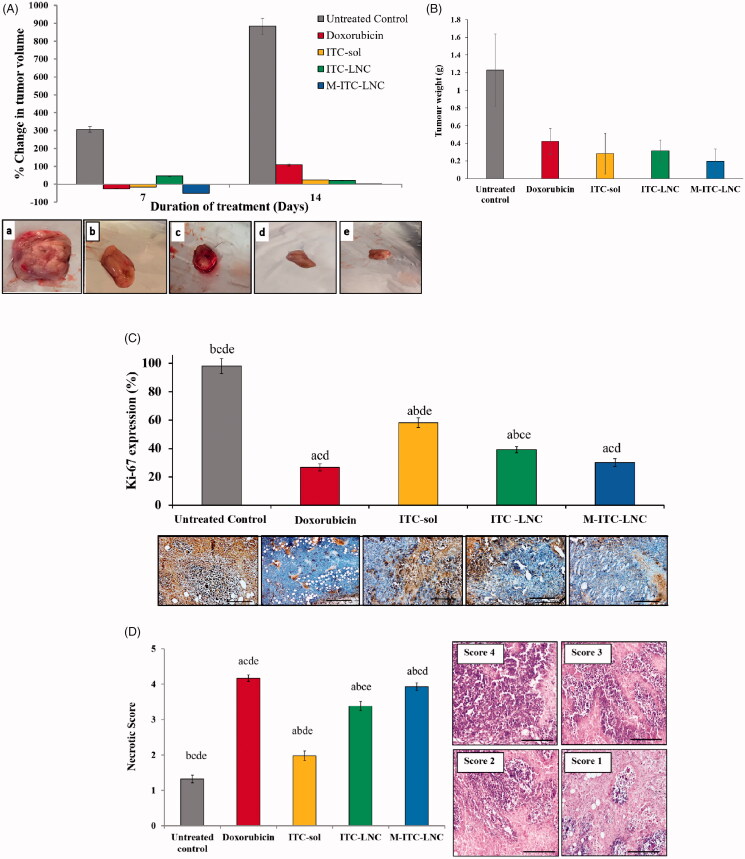
Antitumor efficacy of test formulations in comparison with untreated control and doxorubicin (Dox) 14 days post i.p. treatment of Ehrlich-induced mammary tumors in mice. (A) Percent change in tumor volume relative to pretreatment volume and digital images of excised tumors; (B) weight of excised tumors; (C) immunohistochemical analysis of tumor proliferation using Ki 67 staining. The positive (brown) in tumor sections represents tumor cell proliferation. Error bars represent SD (*n* = 3). (D) Histopathological analysis of tumor necrosis (×40). Necrotic score was determined using a scoring system based on the % necrosis in sections in poorly differentiated tumors as follows: necrotic scores 4, 3, 2, and 1 correspond to >50%, >35%, >25%, and >10% necrosis, respectively; (C, D), ^a^*p*< .05 vs. untreated control; ^b^*p*< .05 vs. Dox; ^c^*p*< .05 vs. ITC solution; ^d^*p*< .05 vs. ITC-LNC; ^e^*p*< .05 vs. M-ITC-LNC and scale bars represent 20 μm.

Results for tumor growth inhibition are shown in Figure 5(A,B). A 310% change in tumor volume in untreated mice at day 7 was significantly (*p* < .05) reduced by Dox and ITC test formulations to less than ±45% (Figure 6(A)). At day 14, untreated mice showed a mean 884% increase in tumor volume. This was significantly (*p*<.05) decreased by Dox and ITC formulations. The tumor growth inhibitory effect of these formulations was in the order M-ITC-LNC > ITC-LNC > ITC-sol. Results were consistent with digital images of the tumors (Figure 6(A)) as well as cytotoxicity and selectivity data (Figure 4(C,D)). The weight of excised tumors was significantly reduced by all treatments, though with insignificant difference due to large variability (Figure 6(B)).

#### Tumor proliferation

Tumor cell proliferation and treatment efficacy can be assessed by the Ki-67 proliferative protein expression determined by immunohistochemical staining of the tumor (Dowsett et al., 2011; Yi et al., 2018). The tumor section of untreated mice showed the maximum Ki-67 expression (Figure 6(C)). Tumor proliferation was reduced ≈72.5% by Dox (*p* < .05). Regarding ITC formulations, ITC-sol significantly inhibited cell proliferation (42% reduction, *p* < .05), an effect induced by Hh pathway inhibition (Wang et al., 2017b). The ITC effect was significantly increased by LNC (≈61%, *p* < .05) and further boosted by MFS-LNC (70%, *p* < .05). The difference in % proliferation reduction by M-ITC LNC and Dox treatments was not significant (*p*> .05).

#### Tumor necrosis

Tumor necrosis by different anticancer treatments can be assessed by a necrotic score estimated semi-quantitatively using H&E stained tumor sections (El-Lakany et al., 2018; Farid et al., 2020). As shown in Figure 6(D), untreated and Dox-treated tumors showed the lowest (1.32) and highest (4.17) necrotic scores, respectively, while ITC-based formulations showed intermediate values. The necrotic effect of ITC-sol was significantly (*p* < .05) increased by ITC-LNC and to a significantly greater extent (*p*<.05) by M-ITC-LNC. Although the difference between the necrotic score of M-ITC-LNC and Dox was relatively small, yet it reached statistical significance. Results were generally consistent with those of antiproliferative activity (Figure 6(C)).

Inhibition of tumor growth and proliferation and induction of tumor necrosis supported the efficacy of ITC against Ehrlich cells-induced mammary carcinoma. ITZ is known to selectively inhibit endothelial cells and the Hh signaling pathway, suppressing angiogenesis and tumor growth and inducing apoptosis (Deng et al., 2020). ITC administration also induces tumor necrosis by decreasing the expression of GLI1 mRNA (Kim et al., 2010).

#### Tumor biomarkers expression

**Figure 7.**
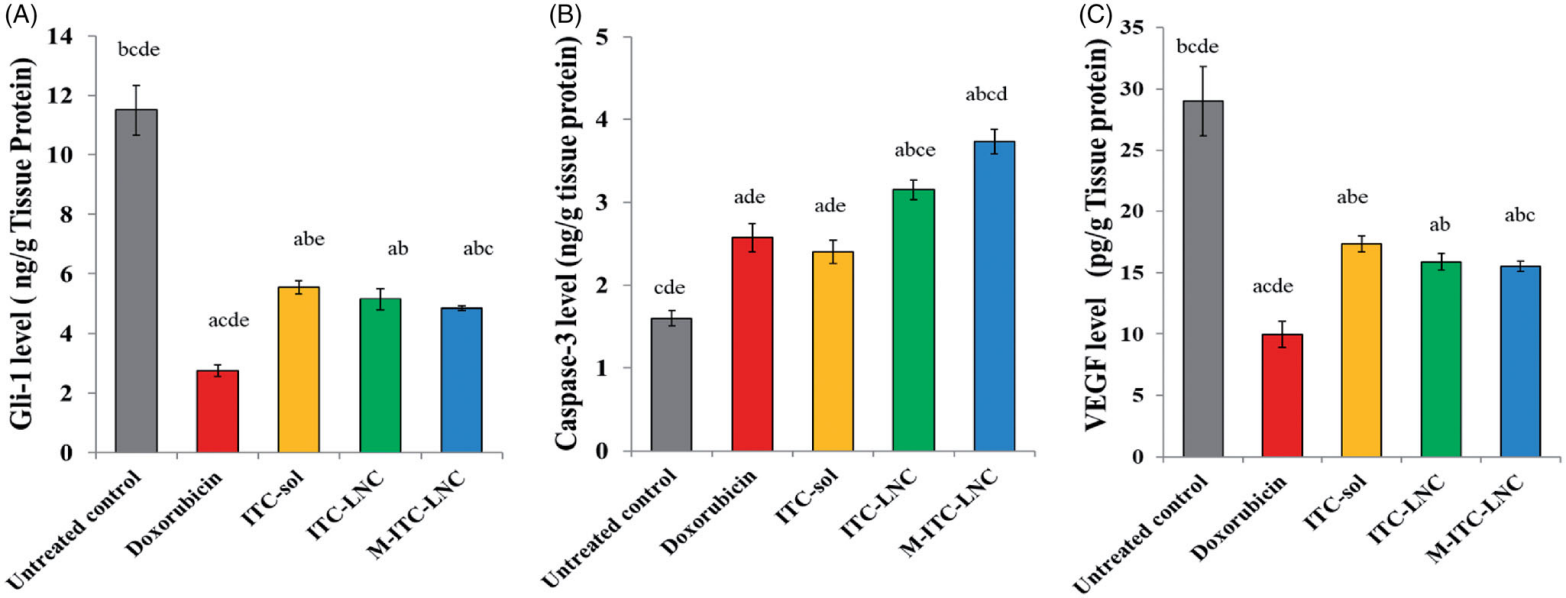
Level of biomarkers in Ehrlich-induced mammary tumor in mice following 14-day treatment with ITC-based formulations in comparison with untreated control and doxorubicin (Dox): (A) Gli-1, (B) caspase-3, and (C) vascular endothelial growth factor (VEGF). ^a^*p*< .05 vs. untreated control, ^b^*p*< .05 vs. Dox, ^c^*p*< .05 vs. ITC-sol, ^d^*p*< .05 vs. ITC-LNC, and ^e^*p*< .05 vs. M-ITC-LNC. Error bars represent SD (*n* = 3).

To validate the *in vitro* and *in vivo* results obtained, the level of three tumor biomarkers, Gli1, caspase-3, and VEGF was assessed quantitatively in the tumor tissue. Gli1 protein is the final transcriptional effector of the Hh signaling pathway, recognized as an indicator of tumor progression and metastasis (Wang et al., 2017a; Xie et al., 2019). As shown in Figure 7(A), tumors of untreated mice had the largest content of Gli1 which was significantly (≈76%, *p* < .05) reduced by Dox. A significant reduction in Gli 1 expression was also induced by ITC-sol (≈51.7%, *p* < .05). ITC is a potent inhibitor of the Hh pathway via a complex signaling cascade culminating into suppression of the Gli family of transcription factors leading to multiple anticancer effects including apoptosis and tumor growth inhibition (Li et al., 2019; Wei et al., 2020). Although LNC did not significantly enhance the ITC effect, the decrease in Gli1 (≈58%) by M-ITC-LNC was significantly different from that of ITC-sol, pointing to an MFS enhancing effect. A structurally related alkylphosphocholine, perifosine, was reported to suppress Hh signaling by inhibiting Gli 1 activation and decreasing its target protein patched 1 expression (Xin et al., 2014).

ITC-induced inhibition of Hh pathway and promotion of cell apoptosis was verified by the increase in caspase-3 level of the tumors in different groups (Figure 7(B)). Caspase-3, an apoptotic biomarker, is activated in response to chemotherapy and is considered a significant indicator for treatment efficacy in breast cancer (Abedin & Barua, 2021). The level of caspase-3 in tumors of all treated groups was significantly higher relative to untreated control. The increase in caspase-3 level by ITC-sol was similar to that of Dox (*p*> .05). Increased caspase-3 expression, an apoptotic mechanism of ITC (Wang et al., 2017b; Wei et al., 2020), was significantly increased (39%, *p* < .05) by LNC and further potentiated by M-ITC-LNC (65%, *p* < .05). Possible MFS-induced bioactivity of LNC, sustained ITC delivery and/or caspase-3 dependent MFS-induced apoptosis of tumor cells (Rybczynska et al., 2001) might account for the results obtained.

VEGF stimulates tumor angiogenesis, an essential requirement for nutrient and oxygen supply and evacuation of metabolic waste (Saman et al., 2020). As shown in Figure 7(C), the highest VEGF level was expressed by untreated control tumors. This was significantly (*p* < .05) lowered by Dox (56%). Significant VEGF suppression (40%) was also induced by ITC-sol known to exert specific and dose-dependent inhibition of endothelial cell proliferation, migration, and tube formation in response to VEGF angiogenic stimulation (Aftab et al., 2011). ITC effect was enhanced by LNC, though the difference in VEGF content reached significance only with M-ITC-LNC (*p* < .05). Inhibition of VEGF expression in cancer cells by MFS could be a contributing factor (Verheij et al., 2001). Anticancer efficacy and tumor levels of Gli 1, caspase-3, and VEFG verified the activity of ITC against breast cancer and provided evidence for the enhancement of such activity by LNC, particularly MFS-modified LNC.

#### Toxicity study

**Figure 8.**
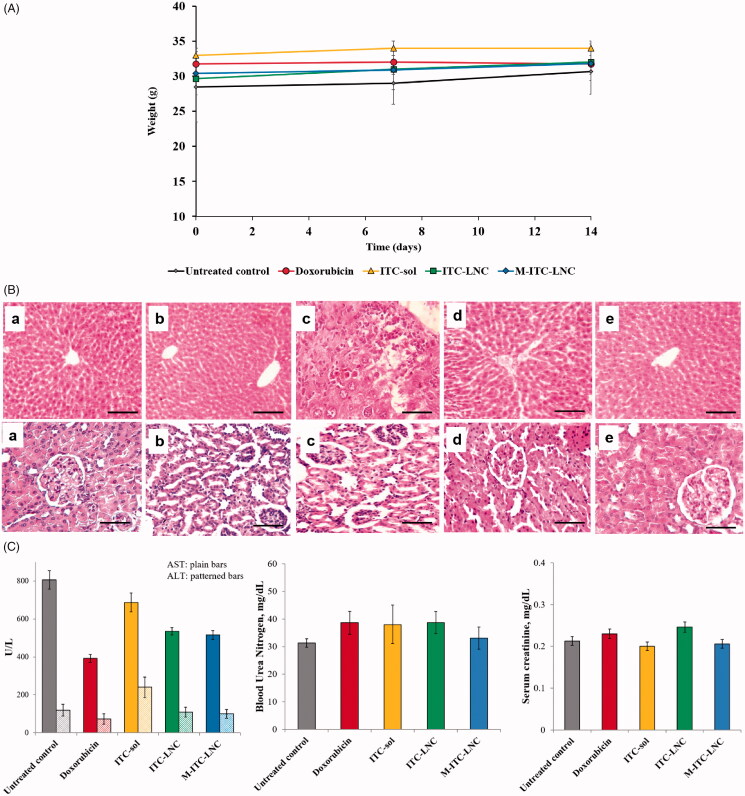
Systemic toxicity in mice post treatment with ITC-based formulations in comparison with untreated control and doxorubicin. (A) Animal weight 7 and 14 days post treatment (*n* = 3–5); (B) histopathological analysis of H&E stained sections of the liver (upper panel) and kidney (lower panel) 14 days post treatment. (a) Untreated mice and mice treated for 14 days with (b) doxorubicin, (c) ITC-sol, (d) ITC-LNC, and (e) M-ITC-LNC. Photomicrographs of liver sections show: (a, b, and e) cords of hepatocytes radiating from a central vein, portal tract endothelial and Von Kupffer cells and hepatocyte cords separated by blood sinusoids and (c, d) dilatation and congestion of portal vein and hepatic artery, bile duct proliferation, and periportal cellular infiltration; photomicrographs of kidney sections (upper panel) show (a, e) normal structure and architecture of renal corpuscles, proximal, and distal tubules, (b–d) widening of tubules with cellular debris and casts within their lumen, congestion of blood vessels, widening of Bowman’s capsule with vacuolation of some cells, cellular debris and casts within their lumen and abnormalities in renal corpuscles. Scale bars correspond to 20 μm; (C) serum levels of the liver enzymes, alanine transaminase (ALT), and aspartate aminotransferase (AST) and the kidney function markers, serum creatinine, and blood urea nitrogen (BUN). Error bars represent SD (*n* = 3).

All mice treated with Dox or the ITC-based test formulations survived the study and appeared healthy. Figure 8(A) indicates that the change in mice weight at days 7 and 14 relative to baseline weight was not significant (*p*> .05), suggesting lack of severe systemic toxicity. As indicated by liver histopathology (Figure 8(B), upper panel), untreated control sections displayed normal features including lobulation with clear outlines and arrangement of hepatocytes in cords radiating from central veins separated by blood sinusoids. These were also observed in the liver section of Dox-treated mice. On the other hand, ITC-sol treatment induced liver injury manifested as reduced cell density, dilatation, and congestion of portal tract veins and the hepatic artery, bile duct proliferation as well as periportal cellular infiltration, in accordance with literature reports (Somchit et al., 2004; Shim et al., 2016). Noteworthy, LNC conferred hepato-protection against ITC-induced injury, which was more pronounced upon MFS modification, corroborating the protective effect of MFS-LNC against disease-induced hepatotoxicity in mice (Eissa et al., 2015). As to kidney histopathology (Figure 8(B), lower panel), sections of untreated control mice (a) displayed normal structure and architecture of renal corpuscles, proximal and distal tubules. Dox treatment (b) induced minor to moderate histological changes including widening of tubules with cellular debris and casts within their lumen and widening of Bowman’s capsule due to potential Dox nephrotoxicity (Sami et al., 2019). Similar but milder changes were observed in the kidney sections of ITC-sol-treated mice (c). In contrast, lipid nanoencapsulation reduced LNC kidney injury (d) and its protective effect was obviously enhanced by MFS modification (e).

Biochemical data (Figure 8(C)) indicated increased serum levels of ALT and AST in untreated mice relative to their reference ranges, 15–84 U/L and 54–300 U/L respectively in mice, suggesting possible cell membrane transport disorder, enzyme outflow, and reduced liver function. Higher ALT and AST values were reported for murine Ehrlich ascites carcinoma relative to normal control values (Saad et al., 2017). The ALT level, more specific to liver injury, was significantly reduced (*p* < .05) by Dox to normal but was elevated two-fold relative to untreated control by ITC-sol, an effect attributed to ITC hepatotoxicity (Shim et al., 2016). This was significantly (*p* < .05) reduced 55% and 59% by ITC-LNC and M-ITC-LNC, respectively. A generally similar trend was observed with AST levels, indicating LNC-induced hepatoprotective effect. Regarding kidney function, BUN, and serum creatinine levels were within the reference ranges of both markers, 0.2–0.8 mg/dL and 30–40 mg/dL, respectively (Hanigan et al., 2001). The observed minor to moderate renal histopathological changes induced by ITC-sol (Figure 7(B), lower panel) might be insufficient to affect the kidney function markers during the ITC-sol 14 day-treatment period. Moreover, biochemical data for ITC-LNC- and M-ITC-LNC-treated mice were consistent with the histopathologically evident kidney protective effect exerted by these formulations, particularly the M-ITC-LNC. Taken together, histopathologic, and biochemical findings pointed to a superior hepatic and kidney protective effect of M-ITC-MFS. Accordingly, improving the biopharmaceutical profile of ITC with MFS-modified LNC enhanced the efficacy and safety of ITC treatment.

## Conclusions

A new miltefosine-modified lipid nanocapsule formulation (M-ITC-LNC) integrating the distinct biopharmaceutical properties of LNC and the structural properties and bioactivity of MFS significantly enhanced the efficacy of ITC against breast cancer. This was verified at both the cellular and molecular levels compared with ITC-sol and ITC-LNC. Apart from increasing ITC anticancer efficacy, M-ITC-LNC enhanced the safety of ITC treatment in a murine mammary pad Ehrlich tumor model. Accordingly, M-ITC-LNC offer promise as a bioactive nano-platform for the delivery of ITC and possibly other chemotherapeutic agents. The challenge of encapsulating drugs having different physicochemical properties into LNC can be overcome by modifying the lipid/surfactant composition of LNC.

## References

[CIT0001] Abdel-Mottaleb MM, Neumann D, Lamprecht A. (2010). In vitro drug release mechanism from lipid nanocapsules (LNC). Int J Pharm 390:208–13. 20149853 10.1016/j.ijpharm.2010.02.001

[CIT0002] Abedin MR, Barua S. (2021). Isolation and purification of glycoglycerolipids to induce apoptosis in breast cancer cells. Sci Rep 11:1298. 33446783 10.1038/s41598-020-80484-x PMC7809038

[CIT0003] Aftab BT, Dobromilskaya I, Liu JO, Rudin CM. (2011). Itraconazole inhibits angiogenesis and tumor growth in non-small cell lung cancer. Cancer Res 71:6764–72. 21896639 10.1158/0008-5472.CAN-11-0691 PMC3206167

[CIT0004] Aghebati-Maleki A, Dolati S, Ahmadi M, et al. (2020). Nanoparticles and cancer therapy: perspectives for application of nanoparticles in the treatment of cancers. J Cell Physiol 235:1962–72. 31441032 10.1002/jcp.29126

[CIT0005] Alhakamy NA, Md S. (2019). Repurposing itraconazole loaded PLGA nanoparticles for improved antitumor efficacy in non-small cell lung cancers. Pharmaceutics 11:685. 10.3390/pharmaceutics11120685 PMC6955961 31888155

[CIT0006] Alonso L, Cardoso EJS, Mendanha SA, Alonso A. (2019). Interactions of miltefosine with erythrocyte membrane proteins compared to those of ionic surfactants. Colloids Surf B Biointerfaces 180:23–30. 31022554 10.1016/j.colsurfb.2019.04.040

[CIT0007] Arndt D, Zeisig R, Eue I, et al. (1997). Antineoplastic activity of sterically stabilized alkylphosphocholine liposomes in human breast carcinomas. Breast Cancer Res Treat 43:237–46. 9150903 10.1023/a:1005798715192

[CIT0008] Ballot S, Noiret N, Hindré F, et al. (2006). 99mTc/188Re-labelled lipid nanocapsules as promising radiotracers for imaging and therapy: formulation and biodistribution. Eur J Nucl Med Mol Imaging 33:602–7. 16450136 10.1007/s00259-005-0007-0

[CIT0009] Carbone C, Martins-Gomes C, Pepe V, et al. (2018). Repurposing itraconazole to the benefit of skin cancer treatment: a combined azole-DDAB nanoencapsulation strategy. Colloids Surf B Biointerfaces 167:337–44. 29684903 10.1016/j.colsurfb.2018.04.031

[CIT0010] Choi YK, Poudel BK, Marasini N, et al. (2012). Enhanced solubility and oral bioavailability of itraconazole by combining membrane emulsification and spray drying technique. Int J Pharm 434:264–71. 22643224 10.1016/j.ijpharm.2012.05.039

[CIT0011] Correia A, Silva D, Correia A, et al. (2018). Study of new therapeutic strategies to combat breast cancer using drug combinations. Biomolecules 8:175. 10.3390/biom8040175 PMC6315516 30558247

[CIT0012] Danaei M, Dehghankhold M, Ataei S, et al. (2018). Impact of particle size and polydispersity index on the clinical applications of lipidic nanocarrier systems. Pharmaceutics 10:57. 10.3390/pharmaceutics10020057 PMC6027495 29783687

[CIT0013] De Abreu Costa L, Henrique Fernandes Ottoni M, Dos Santos MG, et al. (2017). Dimethyl sulfoxide (DMSO) decreases cell proliferation and TNF-α, IFN-γ, and IL-2 cytokines production in cultures of peripheral blood lymphocytes. Molecules 22:1789. 10.3390/molecules22111789 PMC6150313 29125561

[CIT0014] Deng H, Huang L, Liao Z, et al. (2020). Itraconazole inhibits the Hedgehog signaling pathway thereby inducing autophagy-mediated apoptosis of colon cancer cells. Cell Death Dis 11:539. 32681018 10.1038/s41419-020-02742-0 PMC7367825

[CIT0015] Dorlo TPC, Eggelte TA, De Vries PJ, Beijnen JH. (2012). Characterization and identification of suspected counterfeit miltefosine capsules. Analyst 137:1265–74. 22251969 10.1039/c2an15641e

[CIT0016] Dowsett M, Nielsen TO, A'Hern R, et al. (2011). Assessment of Ki67 in breast cancer: recommendations from the International Ki67 in Breast Cancer Working Group. J Natl Cancer Inst 103:1656–64. 21960707 10.1093/jnci/djr393 PMC3216967

[CIT0018] Eissa MM, El-Azzouni MZ, El-Khordagui LK, et al. (2020b). Single oral fixed-dose praziquantel-miltefosine nanocombination for effective control of experimental *Schistosomiasis mansoni*. Parasit Vectors 13:474. 32933556 10.1186/s13071-020-04346-1 PMC7493353

[CIT0019] Eissa MM, El-Moslemany RM, Ramadan AA, et al. (2015). Miltefosine lipid nanocapsules for single dose oral treatment of *Schistosomiasis mansoni*: a preclinical study. PLoS One 10:e0141788. 26574746 10.1371/journal.pone.0141788 PMC4648507

[CIT0020] El-Lakany SA, Elgindy NA, Helmy MW, et al. (2018). Lactoferrin-decorated vs PEGylated zein nanospheres for combined aromatase inhibitor and herbal therapy of breast cancer. Expert Opin Drug Deliv 15:835–50. 30067113 10.1080/17425247.2018.1505858

[CIT0021] El-Sheridy NA, Ramadan AA, Eid AA, El-Khordagui LK. (2019). Itraconazole lipid nanocapsules gel for dermatological applications: in vitro characteristics and treatment of induced cutaneous candidiasis. Colloids Surf B Biointerfaces 181:623–31. 31202972 10.1016/j.colsurfb.2019.05.057

[CIT0022] El Sayed I, Helmy MW, El-Abhar HS. (2018). Inhibition of SRC/FAK cue: a novel pathway for the synergistic effect of rosuvastatin on the anti-cancer effect of dasatinib in hepatocellular carcinoma. Life Sci 213:248–57. 30292831 10.1016/j.lfs.2018.10.002

[CIT0023] Elmeliegy M, Lang I, Smolyarchuk EA, et al. (2020). Evaluation of the effect of P-glycoprotein inhibition and induction on talazoparib disposition in patients with advanced solid tumours. Br J Clin Pharmacol 86:771–8. 31770456 10.1111/bcp.14178 PMC7098856

[CIT0024] Elzoghby AO, Mostafa SK, Helmy MW, et al. (2017). Multi-reservoir phospholipid shell encapsulating protamine nanocapsules for co-delivery of letrozole and celecoxib in breast cancer therapy. Pharm Res 34:1956–69. 28643236 10.1007/s11095-017-2207-2

[CIT0025] Farid RM, Gaafar PME, Hazzah HA, et al. (2020). Chemotherapeutic potential of l-carnosine from stimuli-responsive magnetic nanoparticles against breast cancer model. Nanomedicine (Lond) 15:891–911. 32238029 10.2217/nnm-2019-0428

[CIT0026] Feng D, Peng T, Huang Z, et al. (2018). Polymer–surfactant system based amorphous solid dispersion: precipitation inhibition and bioavailability enhancement of itraconazole. Pharmaceutics 10:53. 10.3390/pharmaceutics10020053 PMC6027051 29695136

[CIT2026330] Fourniols T, Bastien E, Canevat A, et al. (2020). Inhibition of colorectal cancer-associated fibroblasts by lipid nanocapsules loaded with acriflavine or paclitaxel. Int J Pharm 584:119337. 32371002 10.1016/j.ijpharm.2020.119337

[CIT0027] Garcion E, Lamprecht A, Heurtault B, et al. (2006). A new generation of anticancer, drug-loaded, colloidal vectors reverses multidrug resistance in glioma and reduces tumor progression in rats. Mol Cancer Ther 5:1710–22. 16891457 10.1158/1535-7163.MCT-06-0289

[CIT0028] Hanigan MH, Lykissa ED, Townsend DM, et al. (2001). γ-Glutamyl transpeptidase-deficient mice are resistant to the nephrotoxic effects of cisplatin. Am J Pathol 159:1889–94. 11696449 10.1016/s0002-9440(10)63035-0 PMC1867073

[CIT0029] Heurtault B, Saulnier P, Pech B, et al. (2002). A novel phase inversion-based process for the preparation of lipid nanocarriers. Pharm Res 19:875–80. 12134960 10.1023/a:1016121319668

[CIT0030] Hirsjarvi S, Sancey L, Dufort S, et al. (2013). Effect of particle size on the biodistribution of lipid nanocapsules: comparison between nuclear and fluorescence imaging and counting. Int J Pharm 453:594–600. 23747436 10.1016/j.ijpharm.2013.05.057

[CIT0031] Houšť J, Spížek J, Havlíček V. (2020). Antifungal drugs. Metabolites 10:106. 10.3390/metabo10030106 PMC7143493 32178468

[CIT0032] Huynh NT, Morille M, Bejaud J, et al. (2011). Treatment of 9L gliosarcoma in rats by ferrociphenol-loaded lipid nanocapsules based on a passive targeting strategy via the EPR effect. Pharm Res 28:3189–98. 21691892 10.1007/s11095-011-0501-y

[CIT0033] Huynh NT, Passirani C, Saulnier P, Benoit JP. (2009). Lipid nanocapsules: a new platform for nanomedicine. Int J Pharm 379:201–9. 19409468 10.1016/j.ijpharm.2009.04.026

[CIT0034] Kaleağasıoğlu F, Ali DM, Berger MR. (2020). Multiple facets of autophagy and the emerging role of alkylphosphocholines as autophagy modulators. Front Pharmacol 11:547. 32410999 10.3389/fphar.2020.00547 PMC7201076

[CIT0035] Kaleağasıoğlu F, Zaharieva MM, Konstantinov SM, Berger MR. (2019). Alkylphospholipids are signal transduction modulators with potential for anticancer therapy. Anticancer Agents Med Chem 19:66–91. 30318001 10.2174/1871520618666181012093056

[CIT0036] Karim R, Lepeltier E, Esnault L, et al. (2018). Enhanced and preferential internalization of lipid nanocapsules into human glioblastoma cells: effect of a surface-functionalizing NFL peptide. Nanoscale 10:13485–501. 29972178 10.1039/c8nr02132e

[CIT0037] Kim J, Tang JY, Gong R, et al. (2010). Itraconazole, a commonly used antifungal that inhibits Hedgehog pathway activity and cancer growth. Cancer Cell 17:388–99. 20385363 10.1016/j.ccr.2010.02.027 PMC4039177

[CIT0038] Li K, Fang D, Xiong Z, Luo R. (2019). Inhibition of the Hedgehog pathway for the treatment of cancer using Itraconazole. Onco Targets Ther 12:6875–86. 31692536 10.2147/OTT.S223119 PMC6711563

[CIT0039] Lin Y, He X, Zhou D, et al. (2018). Co-delivery of doxorubicin and itraconazole by Pluronic^®^ P123 coated liposomes to enhance the anticancer effect in breast cancers. RSC Adv 8:23768–79. 35540295 10.1039/c8ra03787f PMC9081748

[CIT0040] Lollo G, Matha K, Bocchiardo M, et al. (2019). Drug delivery to tumours using a novel 5-FU derivative encapsulated into lipid nanocapsules. J Drug Target 27:634–45. 30461322 10.1080/1061186X.2018.1547733

[CIT0041] Morille M, Montier T, Legras P, et al. (2010). Long-circulating DNA lipid nanocapsules as new vector for passive tumor targeting. Biomaterials 31:321–9. 19800113 10.1016/j.biomaterials.2009.09.044

[CIT0042] Mouzouvi CRA, Umerska A, Bigot AK, Saulnier P. (2017). Surface active properties of lipid nanocapsules. PLoS One 12:e0179211. 28796777 10.1371/journal.pone.0179211 PMC5552112

[CIT0043] Okeke CI, Eltahan AS, Zhang T, et al. (2017). Co-delivery of itraconazole and docetaxel by core/shell lipid nanocells for systemic antiangiogenesis and tumor growth inhibition. J Biomed Nanotechnol 13:1398–412. 31271127 10.1166/jbn.2017.2428

[CIT0044] Ozaslan M, Karagoz ID, Kilic IH, Guldur ME. (2011). Ehrlich ascites carcinoma. Afr J Biotechnol 10:2375–8.

[CIT0045] Puig-Rigall J, Fernandez-Rubio C, Gonzalez-Benito J, et al. (2020). Structural characterization by scattering and spectroscopic methods and biological evaluation of polymeric micelles of poloxamines and TPGS as nanocarriers for miltefosine delivery. Int J Pharm 578:119057. 31991188 10.1016/j.ijpharm.2020.119057

[CIT0046] Rashidi M, Seghatoleslam A, Namavari M, et al. (2017). Selective cytotoxicity and apoptosis-induction of *Cyrtopodion scabrum* extract against digestive cancer cell lines. Int J Cancer Manag 10:e8633.

[CIT178049] Resnier P, Galopin N, Sibiril Y, et al. (2017). Efficient ferrocifen anticancer drug and Bcl-2 gene therapy using lipid nanocapsules on human melanoma xenograft in mouse. Pharmacol Res 126:54–65. 28159700 10.1016/j.phrs.2017.01.031

[CIT0047] Roger E, Lagarce F, Benoit JP. (2011). Development and characterization of a novel lipid nanocapsule formulation of Sn38 for oral administration. Eur J Pharm Biopharm 79:181–8. 21303693 10.1016/j.ejpb.2011.01.021

[CIT0048] Rybczynska M, Spitaler M, Knebel NG, et al. (2001). Effects of miltefosine on various biochemical parameters in a panel of tumor cell lines with different sensitivities. Biochem Pharmacol 62:765–72. 11551522 10.1016/s0006-2952(01)00715-8

[CIT0049] Saad EA, Hassanien MM, El-Mezayen HA, Elmenawy NM. (2017). Regression of murine Ehrlich ascites carcinoma using synthesized cobalt complex. Med Chem Commun 8:1103–11. 10.1039/c6md00618c PMC6072360 30108821

[CIT0050] Saman H, Raza SS, Uddin S, Rasul K. (2020). Inducing angiogenesis, a key step in cancer vascularization, and treatment approaches. Cancers 12:1172. 10.3390/cancers12051172 PMC7281705 32384792

[CIT0051] Sami MM, Ali EA, Galhom RA, et al. (2019). Boswellic acids ameliorate doxorubicin-induced nephrotoxicity in mice: a focus on antioxidant and antiapoptotic effects. Egypt J Basic Appl Sci 6:10–24.

[CIT0052] Sawasaki M, Tsubamoto H, Nakamoto Y, et al. (2020). S-1, oxaliplatin, Nab-paclitaxel and itraconazole for conversion surgery for advanced or recurrent gastric cancer. Anticancer Res 40:991–7. 32014944 10.21873/anticanres.14033

[CIT0053] Shim JS, Li RJ, Bumpus NN, et al. (2016). Divergence of antiangiogenic activity and hepatotoxicity of different stereoisomers of itraconazole. Clin Cancer Res 22:2709–20. 26801248 10.1158/1078-0432.CCR-15-1888

[CIT0054] Somchit N, Norshahida AR, Hasiah AH, et al. (2004). Hepatotoxicity induced by antifungal drugs itraconazole and fluconazole in rats: a comparative in vivo study. Hum Exp Toxicol 23:519–25. 15625777 10.1191/0960327104ht479oa

[CIT0055] Szwed M, Torgersen ML, Kumari RV, et al. (2020). Biological response and cytotoxicity induced by lipid nanocapsules. J Nanobiotechnol 18:5. 10.1186/s12951-019-0567-y PMC6943936 31907052

[CIT0056] Tsubamoto H, Ueda T, Inoue K, et al. (2017). Repurposing itraconazole as an anticancer agent. Oncol Lett 14:1240–6. 28789339 10.3892/ol.2017.6325 PMC5529765

[CIT0057] Uzunova V, Tzoneva R, Stoyanova T, et al. (2019). Dimethylsphingosine and miltefosine induce apoptosis in lung adenocarcinoma A549 cells in a synergistic manner. Chem Biol Interact 310:108731. 31265827 10.1016/j.cbi.2019.108731

[CIT0058] Van Blitterswijk WJ, Verheij M. (2013). Anticancer mechanisms and clinical application of alkylphospholipids. Biochim Biophys Acta 1831:663–74. 23137567 10.1016/j.bbalip.2012.10.008

[CIT0059] Verheij M, Ruiter G, Zerp S, et al. (2001). Alkyl-lysophospholipids enhance radiation-induced cytotoxicity and inhibit angiogenesis in vitro. Int J Radiat Oncol Biol Phys 51:155. 10.1016/s0360-3016(00)01476-0 11173135

[CIT0060] Wang B, Yu T, Hu Y, et al. (2017a). Prognostic role of Gli1 expression in breast cancer: a meta-analysis. Oncotarget 8:81088. 29113369 10.18632/oncotarget.19080 PMC5655264

[CIT0061] Wang X, Wei S, Zhao Y, et al. (2017b). Anti-proliferation of breast cancer cells with itraconazole: Hedgehog pathway inhibition induces apoptosis and autophagic cell death. Cancer Lett 385:128–36. 27810405 10.1016/j.canlet.2016.10.034

[CIT0062] Wang Y, Yao Y, Liu H, et al. (2015). Itraconazole can inhibit malignant pleural effusion by suppressing lymphangiogenesis in mice. Transl Lung Cancer Res 4:27–35. 25806344 10.3978/j.issn.2218-6751.2014.11.03 PMC4367708

[CIT0063] Wei X, Liu W, Wang JQ, Tang Z. (2020). "Hedgehog pathway": a potential target of itraconazole in the treatment of cancer. J Cancer Res Clin Oncol 146:297–304. 31960187 10.1007/s00432-019-03117-5 PMC11804422

[CIT0064] Xie H, Paradise BD, Ma WW, Fernandez-Zapico ME. (2019). Recent advances in the clinical targeting of Hedgehog/GLI signaling in cancer. Cells 8:394. 10.3390/cells8050394 PMC6562674 31035664

[CIT0065] Xin Y, Shen X-d, Cheng L, et al. (2014). Perifosine inhibits S6K1–Gli1 signaling and enhances gemcitabine-induced anti-pancreatic cancer efficiency. Cancer Chemother Pharmacol 73:711–9. 24519751 10.1007/s00280-014-2397-9

[CIT0066] Yi Q, Ma J, Kang K, Gu Z. (2018). Bioreducible nanocapsules for folic acid-assisted targeting and effective tumor-specific chemotherapy. Int J Nanomedicine 13:653–67. 29440892 10.2147/IJN.S149458 PMC5798557

[CIT0067] Yosifov DY, Kaloyanov KA, Guenova ML, et al. (2014). Alkylphosphocholines and curcumin induce programmed cell death in cutaneous T-cell lymphoma cell lines. Leuk Res 38:49–56. 24225136 10.1016/j.leukres.2013.10.011

[CIT0068] Zhang L, Liu Z, Kong C, et al. (2018). Improving drug delivery of micellar paclitaxel against non-small cell lung cancer by coloading itraconazole as a micelle stabilizer and a tumor vascular manipulator. Small 14:e1802112. 30444572 10.1002/smll.201802112

[CIT0069] Zhang Y, Huo M, Zhou J, et al. (2010). DDSolver: an add-in program for modeling and comparison of drug dissolution profiles. AAPS J 12:263–71. 20373062 10.1208/s12248-010-9185-1 PMC2895453

[CIT0070] Zhou Z, Luo B, Liu X, et al. (2019). Flavonoid-alkylphospholipid conjugates elicit dual inhibition of cancer cell growth and lipid accumulation. Chem Commun 55:8919–22. 10.1039/c9cc04084f 31270526

[CIT0071] Zulueta Diaz YLM, Ambroggio EE, Fanani ML. (2020). Miltefosine inhibits the membrane remodeling caused by phospholipase action by changing membrane physical properties. Biochim Biophys Acta Biomembr 1862:183407. 32628918 10.1016/j.bbamem.2020.183407

